# Supplementary material for: Mechanistically informed machine learning links non-canonical TCA cycle activity to Warburg metabolism and hallmarks of malignancy
Source: PLoS Comput Biol. 2025 Dec 18;21(12):e1013384. doi: 10.1371/journal.pcbi.1013384 (PMC12747436; doi:10.1371/journal.pcbi.1013384)
Supplement: Attachment — Submitted filename: Review letter.pdf [file pcbi.1013384.s019.pdf]

Milan, Novembre 20th 2025

Dear Editors,
Please find enclosed the revised version of the manuscript *"Mechanistically informed machine learning links non-canonical TCA cycle activity to Warburg metabolism and hallmarks of malignancy"* by Lin et al., to be considered for publication in PLOS Computational Biology.

First of all, we would like to congratulate the editors and reviewers for the remarkably high level of the reviews we received. The comments stimulated us to revise several aspects of the work, going well beyond the strictly required changes (which have all been fully addressed).

Although the manuscript has been significantly revised (as evident from the changes marked in blue), the core methodology remains fully intact and all main findings are confirmed. Only some technical aspects of the machine learning module were refined, resulting in more robust results, with some improvements in statistical performance.

Below we summarize the key revisions introduced:

- In response to valuable suggestions from Reviewer #1, we substantially **revised the machine learning module**. In particular, we:
  - grouped genes into functional families to avoid arbitrary selection among correlated features,
  - adopted a multi-target regression framework to predict both metrics jointly,
  - replaced Random Forest with XGBoost for feature selection to ensure consistency with the SHAP analysis, and extended SHAP interpretation to ElasticNet, which contributes to the unified feature panel.

- We added a **new validation section**, applying our flux-based metrics to an independent dataset of tumor/normal spatial transcriptomics to show that they correctly capture increased non-canonical TCA activity in tumor regions.

- We **accounted for confounding variables** (proliferation, histology, TP53 status) in the correlation analysis with DepMap dependency scores, thereby improving robustness and revealing a new gene tightly associated with Warburg-like phenotypes.

- We added an **introductory methodological section within the Results** to facilitate a smoother and more autonomous reading of the main findings.
- We **expanded the Discussion** section to explore the potential role of the non-canonical TCA cycle from an evolutionary perspective and to outline possible validation strategies for our results

We believe that the revisions we implemented have significantly improved both the quality and the clarity of the manuscript.

A point-by-point response to all reviewer comments is provided in the following..

Sincerely,

Chiara Damiani, on behalf of all authors

Reviewers' comments:

Reviewer's Responses to Questions

Comments to the Authors:
Please note here if the review is uploaded as an attachment.

Reviewer #1: Summary
The submitted manuscript describes a study of the newly discovered Non-canonical TCA Cycle (Arnold Cycle) by a combination of constrained-based metabolic modeling and machine learning to identify transcriptomic signatures and marker genes of activity. Since the Arnold Cycle was described only very recently, the authors contribution is novel and interesting for a broader interdisciplinary audience and aims a generating testable hypotheses via in silico approaches in a classical systems biology cycle.
The manuscript is well written, results are well depicted with illustrations and reproducibility is ensured via a open-source repository including code for in silico approaches. Beside these strong points, I see one critical point and additional major as well as minor points (outlined below). The critical point of the study, in my view, is the fragility of the two-step approach without external or experimental validation of the first step where a newly developed metabolic is leveraged to predict pathway activity. As the authors themselves, this is crucial for the second step to predict other marker genes and overall results look fragile towards modelling choices and decisions in the first step. Hence, I suggest that authors outline or even apply a strategy to externally validate the first step gaining confidence and reliability of overall findings.

We thank the reviewer for their careful and constructive evaluation of our work. We greatly appreciated the overall positive tone of the report and, in particular, the insightful suggestion regarding feature aggregation, which proved especially helpful in improving the robustness and interpretability of our machine learning analysis.

Below, we provide detailed responses to each comment.

Critical
Study builds on two steps with predictions without external/experimental validation of the modeling and method. The Authors in the last paragraph acknowledge that metabolic modeling in the first step is crucial for analysis but do no outline or apply a strategy to validate their approach/results before applying ML in the second step. Further, they state that denoising of the transcriptome was necessary which additionally shows that there is a degree of fragility in the two-step approach.

We thank the reviewer for highlighting this important point. We fully agree that an external validation of the flux-based metrics used in the first step is essential, given that these metrics serve as the foundation for the downstream machine learning analysis. Following this very constructive suggestion, we have now incorporated an explicit validation step using an independent dataset of tumor–normal interface samples, where biological expectations regarding metabolic behavior are well established.

In particular, we added a **new section at the beginning of the Results** (now Section 2.2) and a new Figure (Fig. 2) in which we apply our flux-derived metrics to **spatial transcriptomics data from clear cell renal cell carcinoma (ccRCC)** and adjacent normal tissue (Maspero et al., 2024). These data provide a biologically grounded setting in which proliferative tumor regions are expected to display increased non-canonical TCA activity relative to normal tissue. Our metrics successfully recapitulate this pattern, thus providing external support for their biological relevance and strengthening the overall robustness of the two-step framework.

Main
- The benefit of Arnold Cycle unclear: Is it Speed? Cost-Efficiency? Oxygen limit/Warburg … -> Please, address this more in Introduction and Discussion in the sense of “nothing makes sense except in the light of evolution” there must be hypotheses about the necessity of this metabolic route.

We thank the reviewer for raising this foundational question regarding the potential adaptive role of the Cit-Mal Cycle (formerly referred to as the Arnold Cycle).

While we fully agree that understanding the potential selective advantages of this alternative metabolic wiring is essential, we believe that attributing a specific evolutionary benefit to any single metabolic configuration requires appropriate caution. Our flux simulations have indeed showed that the Cit-Mal Cycle is just the tip of the iceberg of a comprehensive rewiring of the overall metabolic network. Indeed, as extensively recognized in systems biology, canonical pathway diagrams, while

highly useful for teaching and conceptual organization, represent simplified views of a highly dynamic and interconnected metabolic network. In practice, individual reactions often participate in multiple pathways, and fluxes can be reversed or rerouted depending on cellular state, signaling context, and environmental constraints.

Nevertheless, the repeated and selective engagement of the Cit-Mal cycle across specific tumor types and cell states suggests that this configuration may confer context-dependent advantages. Recent studies have proposed several non-mutually exclusive hypotheses to explain its biological relevance, including:

- enhancing cytosolic acetyl-CoA availability for lipid biosynthesis and epigenetic regulation;
- contributing to redox homeostasis by modulating NAD^+/NADH balance;
- bypassing mitochondrial steps under hypoxia or Warburg-like conditions;
- retaining reduced carbon to support anabolic precursor pools.

Our results support and extend these hypotheses: we observe that Cit-Mal engagement correlates with increased glycolytic activity and is enriched in cancer cell lines displaying hallmark traits such as invasiveness, angiogenesis, and stemness. These findings suggest that the selective advantage of this non-canonical route may stem not from a single function, but from a convergence of multiple, synergistic benefits. We have now expanded the Introduction and Discussion sections to better contextualize possible biological rationales for its engagement.

Specifically, in the Introduction section (lines 15-17) we specified that we aim to investigate the potential selective advantages that this alternative metabolic route may confer to cancer cells.

Whereas in the Discussion section (lines 456-463) we have added this paragraph to explain the possible metabolic advantages of this pathway:

"*Taken together, citrate rerouting and the associated Warburg-like metabolic phenotype suggest that the non-canonical TCA cycle is not merely an auxiliary pathway within central carbon metabolism, but rather part of a broader reorganization of the metabolic network. This reprogramming of the overall metabolic network likely reflects selective pressures to optimize carbon and redox economy in response to specific cellular and environmental contexts [1, 14, 15]. By retaining reduced carbon instead of oxidizing it to CO2, the cycle may preserve vital intermediates for lipid, nucleotide, and amino acid synthesis. Similarly, regeneration of cytosolic NAD+ and a reduced mitochondrial NADH output collectively lessen the need for oxygen-dependent NADH reoxidation, thus enabling anabolic growth under low-oxygen conditions.*"

Moreover we have rephrased the paragraph on epigenetics (lines 464-497) to better explain that our results motivate the hypothesis that Cit-Mal Cycle could influence epigenetic regulation and cell fate decisions through coordinated metabolite availability. The new paragraph is reported in the following:

"At the transcriptional level, our machine-learning module identifies a set of genes associated with non-canonical TCA cycle activity that is enriched for programs linked to embryonic development and

dedifferentiation. This pattern is consistent with experimental evidence from Arnold-Jackson et al. [1], who implicated the Cit-Mal Cycle in cell state transitions during developmental progression. Such transitions are tightly coupled to chromatin plasticity and epigenetic regulation: dynamic histone and DNA modifications, together with changes in higher-order chromatin organization, are now recognized as central determinants of cell fate and identity during development and reprogramming [48]. Similar principles apply to tumors, where epigenetic plasticity and chromatin disruption can reconfigure cell identity, enabling aberrant fate transitions that contribute to multiple hallmarks of cancer [49]. In line with this view, previous studies have shown that ACLY and the mitochondrial citrate transporter SLC25A1 contribute to the maintenance of cancer stem cell–like properties: knockdown of ACLY suppresses self-renewal capacity and tumorsphere formation in models of NSCLC, HCC, and breast cancer [50, 51], whereas SLC25A1 overexpression in NSCLC and liver cancer stem cells promotes self-renewal, enhances the expression of canonical stemness markers, and is required for proper mitochondrial function [52, 53].

Building on this, and although our model does not explicitly capture epigenetic regulation, we hypothesize that the rerouting of citrate we observe may confer not only metabolic and biosynthetic advantages, but also an epigenetic one. On one side, ACLY-derived acetyl-CoA supports histone and protein acetylation. On the other hand, the citrate-to-αKG branch supplies αKG, a critical cofactor for αKG-dependent dioxygenases involved in histone and DNA demethylation. Coordinated control of flux through these two branches could therefore jointly tune acetylation and methylation dynamics on chromatin, shaping gene-expression programs associated with malignant plasticity in cancer. Previous studies have shown that cytosolic IDH1-derived αKG modulates histone methylation patterns to control lineage commitment, such as in brown adipocyte differentiation, where αKG suppresses adipogenesis by reducing H3K4me3 at key developmental gene promoters [54]. Moreover, mutations in IDH1 and IDH2, recurrently observed in gliomas and acute myeloid leukemias, lead to the neomorphic production of 2-hydroxyglutarate (2HG), a competitive inhibitor of αKG-dependent dioxygenases. This aberrant metabolite impairs histone demethylation, resulting in persistent repression of differentiation-associated genes and maintenance of a progenitor-like transcriptional state [55].”

We hope this addition addresses the reviewer’s point and helps clarify how our findings contribute to the growing understanding of this pathway’s biological significance.

- “we trained regression models to predict both Cycle Propensity and Cycle Flux Intensity” -> In parts of the manuscript it is ambiguous that in fact fully independent ML-models were trained for each target variable. Please, make sure to clearly distinguish from Multi-Task Learning/Transfer-Learning approaches where a simultaneous optimization is pursuit. Perhaps, discuss why such approaches have not been used.

We thank the reviewer for pointing out this important ambiguity. We agree that the original manuscript was misleading, as we had described a multi-output setup while, in practice, training independent models for each target. Conceptually, a multi-task formulation better reflects our goal, since Cycle Propensity and Cycle Flux Intensity are conceptually related and based on the same underlying flux distributions.

We thus revised our pipeline to adopt a **multi-task regression strategy**, simultaneously predicting both targets within a single model. This required adapting our SHAP implementation, since native

support for multi-output regressors—such as XGBoost with the multi_strategy="multi_output_tree" option—is not currently compatible with SHAP's TreeExplainer (which provides highly efficient SHAP value computation for tree-based models). To overcome this limitation, we used SHAP's model-agnostic KernelExplainer, which approximates SHAP values via sampling, allowing correct attribution for both outputs .

As a result, all regression models were **retrained in a multi-task setup**, and all feature selection and SHAP interpretation steps were updated accordingly. These changes are reflected in the revised Methods (Section 4.6.4 - 4.6.5) and Results (Section 2.5), and all affected figures have been updated.

- Highly correlated features (genes) can severely hamper feature importance analysis with different sensitivity towards this issue across the used methods. Hence, I would suggest aggregating gene clusters of very high correlated genes before training models and performing importance analysis to increase robustness.

We are sincerely grateful for this insightful suggestion. Although it may seem straightforward in hindsight, we had not originally considered this strategy, and it proved extremely useful to improve the robustness of our model. Following the reviewer's advice, we introduced a gene clustering step to aggregate highly correlated genes prior to feature selection and model training.

Specifically, we grouped highly correlated genes using a graph-based aggregation approach. Pairwise Pearson correlations were computed across all genes in the filtered expression matrix, and a signed mutual k-nearest neighbor graph was constructed by linking genes that were reciprocally among each other's top positively correlated neighbors. Connected components in this mutual graph were then defined as co-expression modules. For each module, we PCA and used the PC1 as the representative feature.

This strategy:

- improved the robustness of feature selection across models and folds, reducing redundancy and enabling a more precise identification of informative genes.
- It also increased the overlap between ElasticNet and XGBoost outputs: we identified 322 stable features from the MultiTask ElasticNet model, 186 from the multi-output XGBoost model, and 72 genes that were consistently selected by both methods. This overlap facilitates biological interpretation and consistency of the downstream SHAP-based analysis.

The technical details of the procedure and its integration in the pipeline are now thoroughly described in Section~4.6.2 and 4.6.3 of the revised manuscript. For the sake of convenience, we report the new methodology paragraph also here:

"To mitigate the impact of highly correlated features during feature selection, we implemented a graph-based gene aggregation strategy prior to model training. Pairwise Pearson correlations were computed across all genes in the filtered expression matrix, and a signed mutual k-nearest neighbor graph (k = 5) was constructed using positive correlations (r ≥ 0.68). Two genes were connected if each ranked the other among their top k positively correlated neighbors, and connected components in this mutual graph were then defined as clusters of highly co-expressed genes."

“Principal component analysis (PCA) was subsequently applied to each co-expression module identified in this way, using only the training data. For each module, the first principal component (PC1) was retained as its representative feature. Validation and test data were then projected onto the PC1 loadings derived from the training set to ensure consistency across folds.”

- I do not understand the choice of XGBoost in addition to the prior used RandomForests. Please, motivate more why XGBoost + SHAP are used for the final models and which benefit they have over RandomForest + feature importance.

We thank the reviewer for this comment. In the revised manuscript, we replaced Random Forests with XGBoost as the main non-linear learner for both feature selection and interpretation. Although both models are reasonable options, XGBoost consistently achieved higher predictive performance on the full gene-expression matrix. Once additional computational resources became available, the computational burden of training boosted-tree models on tens of thousands of features was no longer prohibitive, allowing us to adopt XGBoost throughout the pipeline.

Compared with the global and often unstable feature-importance scores from Random Forests, SHAP applied to XGBoost provides fine-grained, cell-line–specific attributions. This level of local interpretability was essential for our study, as it enables quantifying the contribution of each selected gene in each individual cell line, thereby identifying context-dependent drivers of the two cycle-activity metrics.

As in the original submission, SHAP was computed after retraining the model on the compact set of features consistently selected across test folds. This strategy enhances interpretability while minimizing noise from weakly informative or unstable predictors.

We now provide an explicit justification for this choice and discuss its advantages in terms of interpretability and comparability across algorithms (Section 2.5, lines 307–312; Section 4.6.7, lines 801–809). We report the original paragraph below:

“To interpret the two cycle-activity metrics at single-gene resolution for the stably selected genes, we use SHAP values to derive local, cell-line–specific attributions, thereby identifying the most informative genes and quantifying their contributions in each line. This approach provides not only a global ranking of predictive genes, but also a map of which genes are decisive in which cell lines, thereby guiding the prioritization of targeted wet-lab hypotheses (joint gene × cell-line selection).”

“To quantify the local contribution of individual genes across cell lines, we computed SHAP values for both MultiTask ElasticNet and multi-output XGBoost models refitted on the subset of consistently selected genes identified across folds (no PCA applied to preserve single-gene interpretability). This approach ensured that interpretability remained focused on transcriptional predictors with demonstrated relevance, rather than being diluted by thousands of genes with negligible influence on model output, while also improving computational efficiency. Moreover, since the predictive models were originally trained on co-expression modules, refitting them on the selected genes enabled SHAP to provide interpretable, cell line–specific attributions at single-gene resolution.”

- Details on train-test-valid splits. The authors perform a cross-validation strategy and I appreciate that

robustness is checked via inner and outer-folds. However, information on how the data is split into training, valid and a final test set is scarce. Further, performance metrics should be reported also on the training set to see the degree of over/underfitting. Lastly, I suggest considering a splitting strategy on the cell line level by reserving e.g. 10% of the >500 cell lines as test-set to check the generalizability of the model.

We are grateful for this suggestion, which helped us improve the clarity and completeness of the evaluation section. While our original framework already employed a rigorous cross-validation strategy that meets the suggested criteria, we acknowledge that these details were not sufficiently emphasized in the initial version.

In the revised manuscript, we now describe our nested cross-validation procedure more explicitly in the Methods section (Section 4.6.3, lines 724-729), which we also report in full below for clarity:

"The dataset was randomly shuffled before partitioning and divided into 72% for training, 18% for validation, and 10% for independent testing. We employed a nested cross-validation strategy comprising 10 outer folds and 5 inner folds. The inner loop was used for hyperparameter optimization, while the outer loop provided an unbiased evaluation of predictive performance and enabled the assessment of feature selection stability across folds."

In response to the reviewer's request, we now also report training set performance metrics (from the inner folds) alongside the test performance, allowing for direct assessment of over/underfitting (see Supplementary Figs. S1–S2). Additionally, we include a learning curve analysis for both MultiTask ElasticNet and multi-output XGBoost models (Supplementary Figs. S3–S4), evaluating performance across progressively larger fractions of the training data. The learning curves demonstrate consistent performance gains and stable generalization, reinforcing the robustness of our predictive models.

Regarding the suggestion to reserve approximately 10% of the cell lines as a fixed or group-level hold-out test set, we acknowledge that such a strategy is appropriate when the primary objective is to evaluate the predictive generalization of a model to cell lines or tumor types that were not observed during training. However, this is not the purpose of the modelling framework developed in this work. Our goal is not to build a deployable predictor of non-canonical TCA cycle activity, but to use machine learning as a feature-discovery tool to identify transcriptional programs associated with this metabolic phenotype and to generate biologically grounded hypotheses for downstream wet-lab validation.

Although a group-level hold-out strategy could, in principle, be informative in contexts where predictive generalization is the primary focus, its interpretation would be less straightforward in our setting. Cancer is highly heterogeneous and transcriptionally plastic, and it is therefore plausible that different tumor lineages engage non-canonical TCA cycle activity through distinct regulatory programs. Under such conditions, lower performance on completely unseen tumor types may not necessarily reflect insufficient model generalization, but rather capture genuine biological divergence in how this metabolic pathway is regulated across tumor contexts. Conversely, excluding an entire lineage from the training pool would prevent the model from learning lineage-specific regulatory signals that may be relevant for feature-discovery purposes. For these reasons, we consider a lineage-level hold-out evaluation unlikely to provide additional insight for the scientific objectives of this study, and we therefore prioritize a nested cross-validation strategy across all available cell lines, which we consider better aligned with the inferential goals of this study.

Minor
- Please, provide line-numbers in the manuscript PDF. This makes giving feedback easier.

Line numbers have now been included in the revised manuscript.

- Give some crucial method details in result part to understand approach without going to Method section or change overall order (Method section before Result)

We have added Section 2.1 in the Results, where the overall approach is presented, and we report it in full below:

"We conceived a two-step computational strategy integrating a metabolic modeling and a machine learning module to dissect the activity of the Cit-Mal Cycle across cancer samples. The strategy is schematized in Fig. 1 and briefly summarized in the following.

The metabolic modeling module employs state-of-the-art constraint-based modeling techniques to infer the activity of the Cit-Mal Cycle directly from gene expression data [2, 5]. Constraint-based models represent cellular metabolism as a network of biochemical reactions subject to stoichiometric mass balance and physicochemical constraints, such as minimal and maximal reaction fluxes [6]. These flux boundaries can be personalized using omics data—such as transcriptomic or proteomic profiles—to reflect the molecular context of individual samples [7]. In this study, we tailored the boundaries of the generic core metabolic network ENGRO2.2 (Sections 4.2 and 4.3) based on the transcriptomes of 513 cancer cell lines cultured under standardized conditions. Specifically, we computed Reaction Activity Scores (RAS) from gene expression using Gene–Protein–Reaction (GPR) rules [8], and used these scores to modulate the upper bounds of each reaction's feasible flux, following the same strategy described in [2, 9].

Each reconstructed model defines the space of feasible steady-state flux distributions for a specific cell line. While classical CBM approaches typically select a single flux vector by optimizing a biological objective (e.g., biomass yield), flux sampling has become an established alternative for systematically exploring the entire solution space consistent with network structure and molecular constraints [10–12]. Accordingly, we employed the corner-based sampling algorithm proposed in [13] to generate thousands of feasible flux distributions per model (Section 4.4).

To this end, we devised two complementary metrics to quantify Cit-Mal Cycle activity from the sampled flux distributions: (i) Cycle Propensity, defined as the fraction of sampled steady-state flux distributions in which all three hallmark reactions (SLC25A1, ACLY, MDH1) are simultaneously active. This metric is dimensionless, ranges from 0 to 1, and reflects the likelihood that the cycle operates under the given constraints; (ii) Cycle Flux Intensity, defined as the average flux through the bottleneck reaction in states where the cycle is active. This metric is reported in arbitrary units. Although the scale is numerically similar to conventional flux units (e.g., mmol/gDW/h), the use of

relative transcriptomic constraints prevents absolute interpretation. Therefore, we explicitly treat these values as arbitrary units.

The machine learning module uses these in silico–derived labels as training targets for supervised regression models designed to predict Cycle Propensity and Cycle Flux Intensity from non-metabolic gene expression data. After model training, we applied feature selection to identify robust transcriptional predictors of Cit-Mal Cycle activity, thereby revealing broader programs potentially regulating or co-occurring with its engagement. Finally, we employed SHAP analysis to quantify the contribution of individual genes to model predictions, providing a biologically interpretable link between transcriptional features and Cit-Mal Cycle activity."

-> Examples: (1) what are main and necessity updates of ENGRO2 -> 2.2? Clearly state that this model is new and not tested in other studies.

We have added the rationale for the updates introduced in ENGRO, together with a brief description of the additional pathways incorporated (Section 4.2, lines 585-606). The original paragraph is shown below:

"Compared to the original ENGRO2 network [2], we introduced two improvements and one essential modification. As improvements, we added reactions for galactose and cholesterol uptake, thereby expanding the range of possible nutrients considered in the simulations, and we refined the GPR rules related to amino acid uptake [63]. In the original ENGRO2 model, some uptake reactions were linked to a limited set of transporters, which caused unrealistic growth impairments in certain cell lines. To address this, we extended the GPR associations by including all known amino acid transporters identified in the Recon3D network [62], using OR logical operators to merge them. These modifications increased the biological coverage and precision of the model without directly affecting the present analysis.

In addition to these improvements, one essential modification was required for this study, namely the extension of cytosolic acetyl-CoA production pathways. In the original ENGRO2 model, ACLY was the sole source of cytosolic acetyl-CoA. Because this metabolite is required for fatty acid synthesis and thus biomass production, ACLY became an artificially essential reaction, predicted to be active in all sampled flux distributions. However, experimental evidence shows that ACLY is not strictly essential, as its loss can be compensated [64]. To better reflect biological reality, we therefore incorporated literature-supported alternative pathways for cytosolic acetyl-CoA production, which include acetyl-CoA generation from acetate via acyl-CoA synthetase short chain family member 2 (ACSS2) [64], endogenous acetate generation [65], and the acetylcarnitine shuttle, where mitochondrial acetyl-CoA is transferred to the cytosol via carnitine acetyltransferase (CrAT) [66]."

(2) Constrained-based modeling approach: sampling approach, (non)-objective function. The metabolic modeling approach and objective remains unclear without fully reading the method section.

As mentioned earlier, we have expanded Section 2.1 of the Results to provide an overview of the constraint-based modeling approach.

- “This inverse relationship suggests that a higher likelihood of Arnold Cycle engagement does not necessarily correspond to increased metabolic throughput.” -> I suggest rephrasing. “Not necessarily” implies more a neutral and not a strong inverse correlation.

We agree with the reviewer that the previous phrasing was ambiguous and could be interpreted as implying a neutral relationship. We have therefore reworded the sentence to convey the intended meaning more clearly. The revised sentence now reads:

“*This inverse relationship indicates that increased likelihood of Cit-Mal Cycle engagement tends to coincide with reduced metabolic throughput, rather than a proportional enhancement of flux.*”

- You may discuss more the interrelation of the Arnold Cycle, Angiogenesis and Warburg. I guess they are connected via low oxygen requirements.

We thank the reviewer for this insightful suggestion. In the revised manuscript, we have added a dedicated paragraph in the Discussion section (lines 514-525) elaborating on the potential interrelation between Mal-Cit Cycle, angiogenesis, and Warburg effect. The paragraph is reported in full below:

“Interestingly, we also observed enrichment for angiogenesis-related programs. This pattern may reflect a coordinated metabolic and transcriptional configuration favoring hypoxia tolerance. At the metabolic level, flux analysis indicated reduced oxygen requirements in cell lines with higher cycle propensity. In parallel, the enrichment of angiogenesis-related pathways represents a transcriptional program facilitating vascular remodeling to restore oxygen delivery [58]. Together, these observations suggest coordinated metabolic and transcriptional adaptations through which non-canonical TCA activity may support cellular resilience under oxygen-limiting conditions. To the best of our knowledge, no direct mechanistic link has yet been experimentally established between enzymes of the non-canonical TCA cycle and angiogenic signaling in cancer, highlighting a potentially unexplored connection between metabolic adaptation and vascular remodeling that warrants further experimental investigation.”

- I understand the priority to relate DepMap with Cycle Propensity. But relating DepMap with Flux Intensity may also provide some insights and could be included in a supplement.

We thank the reviewer for this suggestion. In response, we have now also performed a DepMap correlation analysis using Cycle Flux Intensity.

- Authors make the relation of citrate-to-alpha-KG with epigenetic regulation and cell fate decision. To which degree is this feasible based on the used metabolic model? Is this aspect even captured by the constrained-based modeling approach?

We thank the reviewer for raising this important conceptual point. We have now clarified this aspect more explicitly in the revised Discussion (lines 464–497). In brief, constraint-based model captures only the metabolic rerouting of cytosolic citrate toward α-ketoglutarate production. The model does not represent the downstream fates of αKG, including its potential involvement in chromatin remodeling reactions or other epigenetic processes. Therefore, the link to epigenetic regulation and cell-fate

decisions should be interpreted as a biologically informed hypothesis supported by prior literature, rather than as a direct output of the modeling framework.

- “In particular, it yielded higher silhouette scores when clustering cell lines based on simulated flux distributions, and also led to significantly better alignment with transcriptome-based clusters defined by metabolic gene expression.” -> Do cell lines really need to form good clusters in the flux distribution space similarly to the transcriptome space? I suggest justifying more this assumption and to include the results of this prior analysis in the supplemental material in order to understand this.

We thank the reviewer for raising this important point. Although our statement may appear as an arbitrary assumption, it actually stems from a year-long effort aimed at understanding a puzzling discrepancy: when clustering cell lines based on simulated flux distributions, we observed virtually no overlap with metabolomics-based clusters. Yet, transcriptomic and metabolomic data — both derived from the same cell lines — showed a moderate degree of consistency in their clustering structure. Given that our fluxes were directly constrained using transcriptomic data, this lack of alignment raised concerns about the robustness of our pipeline.

This observation was particularly surprising because our previous applications of transcriptome-constrained modeling to spatial and single-cell data had yielded coherent and biologically meaningful flux profiles. We therefore hypothesized that the issue could lie in the characteristics of the bulk transcriptomic data, particularly the lack of preprocessing or denoising. This was supported by earlier work of ours (https://doi.org/10.1186/s12859-022-04967-6), which showed that even a single zero expression value in a key reaction can block an entire pathway, strongly affecting growth predictions. In those cases, imputing missing or unreliable values (e.g., using MAGIC) significantly improved flux model predictions and cluster coherence.

Following this reasoning, we applied MAGIC to the bulk transcriptomic data and found that it consistently improved the agreement between flux-derived and transcriptome-derived clusters in this dataset. We are still actively investigating the underlying reasons and evaluating potential alternative strategies. Given the breadth of this work and the fact that it is still ongoing, a full discussion would be beyond the scope of the present manuscript. However, all related analyses are now publicly available in a master’s thesis deposited on Zenodo (DOI: https://doi.org/10.5281/zenodo.17452876), which is now cited in the Methods section.

- “In line with this, preliminary flux sampling experiments using the Recon3D [46] model revealed that both canonical and non-canonical TCA cycles were poorly represented. These results support the use of a core model for our analysis.” -> Similar as above, Authors refer to a prior analysis which is not included in the manuscript or a electronic supplement. I suggest including all of those to foster transparency.

We have now added the preliminary Recon3D flux sampling analyses to the Supplementary Material (Supplementary Fig. S 10).

- Why were weights for objective function sampled with negative and positive values with switching

between maximization and minimization? Why not only maximization?

We thank the reviewer for raising this point. In flux balance analysis, fluxes can assume both positive and negative values, depending on the reaction directionality. Minimizing a reaction's flux can therefore be interpreted as maximizing its flux in the reverse direction. By sampling both positive and negative weights and alternating between minimization and maximization, we ensure a more comprehensive exploration of the solution space—including flux configurations where reactions operate in the reverse direction.

While the algorithmic details are described more extensively elsewhere (see references [12, 13]), we have added an explanatory sentence in the revised manuscript to clarify this conceptual point (lines 660–660).

"In this study, we employed the Corner-based Sampling (CBS) algorithm to sample the vertices of the feasible flux space by iteratively optimizing randomly weighted objective functions [12, 13]. Specifically, we used the implementation proposed by Galuzzi et al. [13] to maximize coverage of the feasible solution space. To this end, weights were assigned to reactions by sampling from a uniform distribution in the range [−1, 1], and the optimization direction (maximization or minimization) was randomly selected at each iteration."

- The overlap between ElasticNet and RandomForest discovered genes (6) is surprisingly little. Filtering and aggregating highly correlated genes should improve this. I suggest having a closer look at those 6 genes and their role.

As mentioned above, after aggregating highly correlated genes, the agreement between the two models improved substantially, and the revised analysis now yields 72 genes shared by both models.

- When applying SHAP for estimating feature importance, the definition of a reference/background data set can be crucial. Please, give information and justification on what is used here. A self-defined set or a randomized set?

We used a randomized background set: specifically, 400 samples were randomly drawn from the training data of each fold (out of 461 total) and used as the SHAP background. This information has now been added to the Methods (Section 4.6.7, lines 819-823).

"Training and evaluation followed the same nested cross-validation and hyperparameter optimization strategy used throughout the study. SHAP values were estimated using the KernelExplainer, with 400 background samples randomly drawn from the training data of each fold (out of 461 total) and were computed on the corresponding outer-test sets to obtain per–cell-line attributions."

Reviewer #3: Review: Lihao et al., investigated how cancer cells leverage metabolic circuits to support phenotypic plasticity and progression, with a particular focus on the non-canonical citrate metabolism. Recognizing the difficulty of inferring compartment-spanning fluxes from existing datasets, they develop a mechanistically informed machine learning framework that integrates constraint-based metabolic modeling with supervised learning. Using non-canonical citrate metabolic

activity metrics derived from over 500 cancer cell lines, they identify transcriptional programs predictive of cell cycle activity. Their approach not only establishes robust transcriptional correlates of citrate metabolic flux but also uncovers broader links between this cycle and metabolic reprogramming, providing new insights into how metabolic flexibility underpins aggressive cancer phenotypes.

1- The manuscript designates the metabolic circuit as the Arnold Cycle. However, I note that the original foundational study on this pathway listed two co–first authors, both of whom should receive equivalent recognition. As such, if the authors wish to propose a new phenotype or formalize a naming convention, the terminology should reflect joint credit (e.g., Arnold–Jackson or Arnold–Jackson–Finely). Alternatively, a more neutral and descriptive designation, such as non-canonical citric metabolism, may be preferable to avoid ambiguity or inequity in attribution.

We thank the reviewer for raising this important clarification regarding terminology and attribution. We agree that referring to the pathway solely as the “Arnold Cycle” does not fully acknowledge the joint contributions of all co–first authors of the foundational study. To avoid ambiguity and ensure equitable recognition, we have revised the manuscript accordingly.

In the updated version, we now adopt the more neutral and biochemically descriptive term **“Cit–Mal Cycle”** when referring to the pathway. In all instances where we cite the original experimental discovery, we explicitly use the formulation **“Arnold–Jackson et al.”**, ensuring that both co–first authors receive equal attribution.

2- Figure 1b comes before Figure 1c in the text. The order should be fixed either in the figure or in the text.

The order of panels in Figure 1 and the corresponding citations in the text have now been corrected.

3- is claimed that:” From these samples, we derived two complementary metrics: Cycle Propensity, defined as the fraction of sampled states in which the three core reactions (SLC25A1, ACLY, MDH1) of the Arnold Cycle are simultaneously active; and Cycle Flux Intensity, measuring the average flux through the cycle’s bottleneck reaction in active configurations (Fig. 1b).” However, Figure 2 doesn’t show this; and the two concepts needs much more clarifications.

We thank the reviewer for this helpful observation. We agree that the previous version of Fig. 1b did not sufficiently illustrate how the two metrics—Cycle Propensity and Cycle Flux Intensity—are computed. In the revised manuscript, we redesigned this panel to include numerical examples and step-by-step illustrations of how each metric is derived from the sampled flux states, thereby greatly improving interpretability. In addition, we introduced a new Section 2.1 in the Results to provide a concise summary of the overall computational framework.

4- Cancer is separated to stages and subtypes and any of the cell lines are assigned to types or subtypes of cancer such as MDA-mb-231 as triple negative metastatic breast cancer with high Warburg phenotype features or MCF7 as non-metastatic hormone positive cell types. These should be tested in the model comparing for example 231 cells as high Warburg phenotype compared to

MCF7 as low Warburg phenotype and normal such as MCF10A. These can be also tested in section 2.2 for Warburg phenotype and oxygen consumption.

We thank the reviewer for this valuable and thoughtful suggestion.

Before addressing the point directly, it is worth emphasizing that the transcriptionally informed constraint-based metabolic modeling strategy used to derive samples cell-specific flux distributions has previously proven effective in capturing meaningful metabolic differences, both across breast cancer cell lines [https://doi.org/10.1371/journal.pcbi.1009337] and between tumor regions [https://doi.org/10.1101/2024.11.28.625842]. It is important to clarify this strategy is inherently designed for cross-sample comparisons, not for estimating absolute metabolic fluxes. This is due to the relative nature of the transcriptomic constraints used to define the solution space, which makes flux values and derived metrics meaningful only when interpreted in a comparative context.

The methodological innovation introduced in this study lies in the design of the two flux-based metrics — Cycle Propensity and Cycle Flux Intensity — aimed at quantifying Cit-Mal Cycle activity from the sampled flux distributions. Also in response to Reviewer 1's comment, we sought to evaluate whether these two metrics are consistent with independent, experimentally derived signatures of Cit-Mal Cycle activity. In particular, the original study by Arnold-Jackson et al. demonstrated that proliferative cells—such as cancer and embryonic stem cells—preferentially activate the Cit-Mal cycle, whereas its engagement diminishes in differentiated and less proliferative cells [ref 2, 21, 22]. We therefore tested whether these flux-derived metrics reproduce the experimentally observed increase in theCit-Mal Cycle activity characteristic of proliferative cell states.
Since the panel of 513 cancer cell lines lacks matched normal controls, we leveraged spatial transcriptomics data from clear cell renal cell carcinoma (ccRCC) and adjacent normal tissue to assess whether our flux-based metrics capture the expected metabolic behavior of proliferative tumor regions. To this end, we computed our flux-based metrics from transcriptionally informed flux distributions previously generated in [https://doi.org/10.1101/2024.11.28.625842] from these spatial profiles, which reproduced the characteristic increase in biomass synthesis flux observed in tumor regions.
As expected, both cycle-specific metrics were significantly elevated in tumor areas, with increased Cycle Propensity ($t = 39.61$, $p = 7.3 \times 10^{-178}$) and Cycle Flux Intensity ($t = 96.30$, $p < 10^{-308}$), indicating enhanced engagement of the Cit–Mal Cycle in cancer cells.
These results are now reported in the new Section 2.2 and new Figure 2, confirming that our flux-derived metrics faithfully recapitulate the preferential engagement of the Cit-Mal cycle in proliferative, tumor-associated regions, in agreement with its experimentally observed activation in rapidly dividing cells.

In response to reviewer 3's suggestion, we examined a subset of three breast cancer cell lines for which the Warburg phenotype has been well characterized in the literature through experimental measurements of nutrient exchange rates. Bearing in mind that our transcriptionally informed flux sampling strategy is not designed to estimate absolute flux values, we focused on the relative ordering of cell lines with respect to their metabolic profiles rather than on quantitative differences in flux rates. In fact, when the same breast cancer lines are analyzed within a pan-cancer dataset, their inferred fluxes may differ from those obtained in a breast-specific dataset. This is an inherent limitation of using relative transcriptomic constraints: the presence of highly dissimilar samples can

compress or skew differences between more similar ones. However, the ordering should be preserved.

Among the cell lines mentioned by the reviewer, MDA-MB-231 is included in our dataset, whereas MCF7 and MCF10A are not available. For this reason, we used two additional breast cancer lines present in our dataset—MDA-MB-453 and MDA-MB-361—for which experimental measurements of glucose consumption and lactate secretion have been reported (refs. 21, 22). Based on these experimental data, both lines exhibit a markedly lower Warburg phenotype compared to MDA-MB-231.

Among the three cell lines under study (MDA-MB-231, MDA-MB-453, and MDA-MB-361), MDA-MB-231 is widely recognized as a prototypical high-Warburg cell line, whereas MDA-MB-453 and MDA-MB-361 display a weaker Warburg phenotype, with lower lactate secretion and glycolytic activity. Consistent with expectations, our simulations showed that MDA-MB-231 had markedly higher non-canonical cycle activity than the other two lines (Cycle Propensity: 0.458 vs 0.367 and 0.365; Cycle Flux Intensity: 15.73 vs 10.74 and 10.93 a.u.). Regarding Warburg-related fluxes, MDA-MB-231 also exhibited slightly higher lactate secretion (5.61 vs 5.17 and 5.13 a.u.) and lactate-to-glucose ratios (0.69 vs 0.67 and 0.65), while oxygen uptake was similar across the three lines (30.75 vs 29.26 and 30.86 a.u.). These modest differences likely reflect the intermediate positioning of breast cancer cells within the broader metabolic landscape captured by our pan-cancer dataset. Nonetheless, within this constrained dynamic range, our simulations reproduced the expected hierarchy of Warburg activity, with MDA-MB-231 exhibiting the most glycolysis-dominant metabolic profile.

This new analysis is reported in Section 2.4 (lines 187-216) and also included below for the reviewer's convenience.

"As we have already emphasized above, the transcriptionally informed flux sampling strategy used to derive our non-canonical TCA cycle metrics has previously proven effective in capturing differences between breast cancer cell lines [2] and between tumor regions, [5]. However, this methodology is not designed to estimate absolute flux values. As a consequence, the predicted flux for a given cell line may vary depending on the broader dataset context. For example, when the same breast cancer cell lines are analyzed within a pan-cancer dataset, their inferred fluxes may differ from those obtained in a breast-specific dataset. This is an inherent limitation of using relative transcriptomic constraints: the presence of highly dissimilar samples can compress or skew differences between more similar ones. Nevertheless, we expect that the relative ordering of cell lines with respect to their metabolic profiles should be preserved. To test this hypothesis and further explore the relationship between Warburg metabolism and non-canonical TCA cycle activity, we focused on a subset of three breast cancer cell lines for which the Warburg phenotype has been well characterized in the literature through experimental measurements of nutrient exchange rates: MDA-MB-231, MDA-MB-453, and MDA-MB-361. Among these, MDA-MB-231 is widely recognized as a prototypical high-Warburg cell line, whereas MDA-MB-453 and MDA-MB-361 display a weaker Warburg phenotype, with lower lactate secretion and glycolytic activity [2, 21, 22].

Consistent with expectations, our simulations showed that MDA-MB-231 had markedly higher non-canonical cycle activity than the other two lines (Cycle Propensity: 0.458 vs 0.367 and 0.365; Cycle Flux Intensity: 15.73 vs 10.74 and 10.93 a.u.). Regarding Warburg-related fluxes, MDA-MB-231 also exhibited slightly higher lactate secretion (5.61 vs 5.17 and 5.13 a.u.) and lactate-to-glucose ratios (0.69 vs 0.67 and 0.65), while oxygen uptake was similar across the three lines (30.75 vs 29.26 and 30.86 a.u.).

These modest differences likely reflect the intermediate positioning of breast cancer cells within the broader metabolic landscape captured by our pan-cancer dataset. Nonetheless, within this constrained dynamic range, our simulations reproduced the expected hierarchy of Warburg activity, with MDA-MB-231 exhibiting the most glycolysis-dominant metabolic profile."

5- DepMap dependency correlations
6- For DepMap dependency correlation analysis, they correlate Propensity with CERES scores genome-wide and highlight WNT/KRAS/PI3K/MAPK patterns. It is critical to multiple-testing control and confounders (lineage, proliferation rate, TP53 status).

We thank the reviewer for these very valuable suggestions, which prompted us to refine and strengthen our DepMap dependency analysis. In the revised manuscript, we now account for key confounders—namely proliferation rate, TP53 status, and histological subtype—by residualizing both Cycle Propensity and CERES scores prior to correlation analysis. Partial correlations between our two cycle-activity metrics and the residualized CERES values were then computed, as detailed in the Methods (section 4.9).

As in the previous version, multiple-testing correction (Benjamini–Hochberg FDR) was applied to all genome-wide dependency analyses. For the three enzymes constituting the Cit–Mal Cycle, we did not apply multiple-testing correction because only three correlations were assessed; this is now stated explicitly in the Methods, as it was not clearly articulated before.

Following Reviewer 1's suggestion, we extended the analysis to both Cycle Propensity and Cycle Flux Intensity. For Cycle Propensity, 28 genes remained significant after FDR correction, including SLC2A1 (GLUT1; glucose transporter 1), which showed the strongest association ($\rho = -0.29$, FDR = 3.1×10^{-3}). This association indicates a stronger glucose dependency in high-propensity cell lines, which is consistent with the Warburg-like phenotype highlighted by our in silico results. In addition, the WNT-signaling–related gene set remained significantly correlated with Cycle Propensity even after removing the confounders, reinforcing the robustness of this association. For Cycle Flux Intensity three significant dependencies were identified.

7- The manuscript remains purely in silico. Even a lightweight orthogonal validation would greatly strengthen the claims:
a) Select 2–3 "high-propensity/low-intensity" lines and 2–3 "low-propensity/high-intensity" lines; measure OCR/ECAR and lactate secretion (Seahorse/biochemical assays) to verify predicted trends;
b) Test sensitivity to ACLY (SB-204990, Bempedoic-acid CoA analog) and SLC25A1 (CTPi), plus IDH1 inhibition (AG-120 in IDH1-WT as control). Show differential viability/flux adaptation consistent with your dependency analyses;
c) Perform limited ^{13}C-glucose/^{13}C-citrate tracing to confirm cytosolic citrate routing via ACO1→IDH1 vs ACLY.

We sincerely thank the reviewer for this insightful suggestion. We fully agree that wet-lab validation represents an important next step, and we share the enthusiasm for experimentally testing the hypotheses generated by our computational framework.

The main aim of the present study, however, was to develop a mechanistically informed computational strategy and to generate testable hypotheses regarding the regulation and functional relevance of the Cit–Mal Cycle in cancer. The breadth of the experimental validation proposed—OCR/ECAR measurements, pharmacological inhibition of ACLY/SLC25A1/IDH1, and ^13C-tracing assays—would require substantial dedicated wet-lab resources and lie beyond the scope of the current computational investigation. Nevertheless, we fully recognize the importance of these experiments and agree that they would provide valuable orthogonal support for the mechanistic insights obtained in silico.

Reflecting this, we have added a dedicated paragraph in the Discussion outlining concrete avenues for future experimental validation, including the very strategies suggested by the reviewer. We believe that articulating these experimental directions strengthens the translational relevance of our findings and provides a clear roadmap for future work. We are grateful to the reviewer for highlighting this opportunity and hope that our expanded discussion will help guide the experimental exploration of this recently discovered pathway in future studies.

The newly added paragraph is reported below:

“The hypotheses emerging from our computational analysis point to several directions for experimental validation. The predicted rerouting of cytosolic citrate toward the ACO1–IDH1 branch represents an intriguing metabolic adaptation that could be directly assessed through isotope tracing experiments, testing whether citrate-derived carbon is preferentially channeled to αKG rather than converted to acetyl-CoA via ACLY. Likewise, the association between non-canonical TCA cycle engagement and a Warburg-like phenotype could be verified by measuring oxygen consumption rate, extracellular acidification rate, and lactate secretion across representative high-propensity/low-intensity and low-propensity/high-intensity cell lines. In this regard, our comparison of simulated flux profiles for breast cancer cell lines with experimentally characterised Warburg phenotypes already provides a preliminary consistency check, showing qualitative agreement between model-predicted Warburg-related flux and experimental observation (Section 2.4). Although our computational framework identifies robust associations between malignancy-associated transcriptional programs and non-canonical TCA activity, it does not resolve causal regulators from downstream effectors. One potential approach to disentangle these relationships would be to measure histone acetylation at promoters of selected genes, since ACLY-derived acetyl-CoA directly contributes to chromatin acetylation; however, such epigenetic data are currently unavailable in the CCLE resource. Nonetheless, the genes identified through our machine learning–based feature selection, together with their SHAP-derived cell line–specific contributions, provide a rational foundation for selecting gene–cell line pairs for targeted experimental validation. Functional perturbation studies—such as gene knockdown or knockout—could help clarify the causal influence of these genes on non-canonical TCA cycle activity and the broader metabolic–transcriptional coupling inferred from our analysis.”

Minor Comments
Throughout: fix small typos (“bas been”, “characterizate”, “cine–specific”, “theWarburg”, “ACLY, promotes”), and a repeated “p = 5.50 < 10−91 / 6.24 < 10−110” formatting, should be “$p < 5.5\times10^{-91}$”, etc.

All issues have now been corrected throughout the revised manuscript.

Figure 1A/B: add units for fluxes (arbitrary units are fine but state “normalized steady-state flux”).

We thank the reviewer for pointing this out. We have now clarified the units used for our metrics in the new Section 2.1 (64-72), as reported in the paragraph below. We have also added explicit units to all flux values in the figure captions of all figures, indicated as arbitrary units (a.u.).

"(i) Cycle Propensity, defined as the fraction of sampled steady-state flux distributions in which all three hallmark reactions (SLC25A1, ACLY, MDH1) are simultaneously active. This metric is dimensionless, ranges from 0 to 1, and reflects the likelihood that the cycle operates under the given constraints; (ii) Cycle Flux Intensity, defined as the average flux through the bottleneck reaction in states where the cycle is active. This metric is reported in arbitrary units. Although the scale is numerically similar to conventional flux units (e.g., mmol/gDW/h), the use of relative transcriptomic constraints prevents absolute interpretation. Therefore, we explicitly treat these values as arbitrary units."

Figure 2A: add sample sizes per lineage; consider rug plots/marginal densities for each axis.

We have implemented all proposed improvements in Figure 2A (now Figure 3A). Specifically, we now report the sample size for each lineage directly in the legend, and we have added both rug plots and marginal kernel density estimates along the top and right axes to better illustrate the distribution of samples.

Figure 3B: report odds ratios and top term counts; consider reducing redundancy with parent-child trimming.

We have replaced the bar plot with a lollipop plot in Figure 3B (now Figure 4B). The updated visualization now includes explicit indicators of both the odds ratio and the gene count for each enriched term, thereby improving clarity. Redundancy among GO terms has also been addressed using REVIGO, which summarizes enrichment results.

SHAP plots: add fold-wise variation bars or stability heatmaps; ensure no metabolic genes slipped into the non-metabolic feature set (list in Supp. Table S1).

We thank the reviewer for this helpful suggestion. We have now quantified SHAP stability across folds and incorporated this information into the revised supplementary material (Supplementary Figures S7–S8).

Regarding feature preprocessing, we confirm that metabolic genes could not appear among the selected predictors, as they were removed during input preprocessing using Ensembl biotype annotations.

Reviewer #2: This is an interesting paper in which the authors use a combination of mechanistic modelling and machine learning to investigate the extent to which the non-canonical TCA cycle is active in approximately 500 cancer cell lines and show how its activity correlates with enrichment in genes associated with the hallmarks of cancer (eg angiogenesis, invasion, stemness). Excitingly, these findings provide new insights into how cancer cells can adapt their metabolism as they become more pathological and could serve as a basis for new targets for treating cancer.

The paper is well-written and logically structured, with the results clearly stated and developed in a logical manner. The methods used are outside my expertise so I am unable to comment on them (and hope that the other reviewers are able to comment on the methodology). From what I understand, the methods used seem to be fairly straight forward, but they are appropriate for the questions of interest and build on the authors' earlier work.

We thank the reviewer for the positive and encouraging comments on our manuscript. We are particularly grateful for the clarity and intellectual honesty with which the reviewer assessed the scope of the work, especially in relation to the methodological aspects. We appreciate the recognition that, even though some techniques fall outside their direct expertise, the overall approach is appropriate, well-grounded, and logically structured.

We are glad that the biological implications of our findings—particularly their potential relevance to understanding cancer progression and identifying therapeutic targets—were considered exciting and impactful.

Have the authors made all data and (if applicable) computational code underlying the findings in their manuscript fully available?
The PLOS Data policy requires authors to make all data and code underlying the findings described in their manuscript fully available without restriction, with rare exception (please refer to the Data Availability Statement in the manuscript PDF file). The data and code should be provided as part of the manuscript or its supporting information, or deposited to a public repository. For example, in addition to summary statistics, the data points behind means, medians and variance measures should be available. If there are restrictions on publicly sharing data or code —e.g. participant privacy or use of data from a third party—those must be specified.

Reviewer #1: Yes

Reviewer #2: Yes

Reviewer #3: **No:**

PLOS authors have the option to publish the peer review history of their article (what does this mean?). If published, this will include your full peer review and any attached files.

If you choose "no", your identity will remain anonymous but your review may still be made public.

Do you want your identity to be public for this peer review? For information about this choice, including consent withdrawal, please see our Privacy Policy.

Reviewer #1: **Yes:** Jan Ewald

Reviewer #2: No

Reviewer #3: **Yes:** mehdi damaghi

[NOTE: If reviewer comments were submitted as an attachment file, they will be attached to this email and accessible via the submission site. Please log into your account, locate the manuscript record, and check for the action link "View Attachments". If this link does not appear, there are no attachment files.]

Figure resubmission:

While revising your submission, we strongly recommend that you use PLOS's NAAS tool (https://ngplosjournals.pagemajik.ai/artanalysis) to test your figure files. NAAS can convert your figure files to the TIFF file type and meet basic requirements (such as print size, resolution), or provide you with a report on issues that do not meet our requirements and that NAAS cannot fix.

After uploading your figures to PLOS's NAAS tool - https://ngplosjournals.pagemajik.ai/artanalysis, NAAS will process the files provided and display the results in the "Uploaded Files" section of the page as the processing is complete. If the uploaded figures meet our requirements (or NAAS is able to fix the files to meet our requirements), the figure will be marked as "fixed" above. If NAAS is unable to fix the files, a red "failed" label will appear above. When NAAS has confirmed that the figure files meet our requirements, please download the file via the download option, and include these NAAS processed figure files when submitting your revised manuscript.

Reproducibility:

To enhance the reproducibility of your results, we recommend that authors of applicable studies deposit laboratory protocols in protocols.io, where a protocol can be assigned its own identifier (DOI) such that it can be cited independently in the future. Additionally, PLOS ONE offers an option to publish peer-reviewed clinical study protocols. Read more information on sharing protocols at https://plos.org/protocols?utm_medium=editorial-email&utm_source=authorletters&utm_campaign=protocols

---

(Remove my information/details)
